# Cell Death, Inflammation and Oxidative Stress in Neurodegenerative Diseases: Mechanisms and Cytoprotective Molecules

**DOI:** 10.3390/ijms222413657

**Published:** 2021-12-20

**Authors:** Anne Vejux

**Affiliations:** Team Bio-PeroxIL, “Biochemistry of the Peroxisome, Inflammation and Lipid Metabolism” (EA7270), Université de Bourgogne Franche-Comté, INSERM, UFR Sciences Vie Terre et Environnement, 21000 Dijon, France; anne.vejux@u-bourgogne.fr

Neurodegenerative diseases are the most common chronic neurological pathologies associated with age, with a major impact on the patient’s quality of life. These pathologies are a heavy medical, social and economic burden, yet there is no causal treatment available. Among the factors contributing to neurodegeneration are cell death caused by different mechanisms (apoptosis, autophagy, reticulum stress, necrosis, necroptosis), high oxidative stress resulting from a disturbed balance between prooxidant and endogenous antioxidant systems, and also inflammation. As treatments for the causes are not available, research has been focused on evaluating molecules of natural or synthetic origin to expand the therapeutic arsenal that could be made available.

The study of the different processes in pathologies that are not neurodegenerative but that are associated with neurodegeneration also advances our knowledge.

In the context of Alzheimer’s disease, it has been shown that energy metabolism can have an impact on the development of the disease. Kubis-Kubiak et al. studied the impact of hyperglycemia or insulinemia on the secretion of the neuronal protein S100B in relation to oxidative stress, nitrosative stress and DNA damage in neurons [1]. This team was able to show that the S100B protein could play a key role in the local toxicity induced by high glucose or insulin concentrations in the early stages of the disease [1]. It would then be interesting to evaluate, as proposed by the authors, the protective mechanisms linked to this protein, in particular by looking at the relationships between S100B and the glucose transporters GLUT1 and GLUT3 in the brain or within the insulin receptor (IR). We might use the S100B protein as a diagnostic marker for the early stages of neuropathological disorders [1]. As the abovementioned authors have suggested, identifying the markers capable of helping in the diagnosis or in following the evolution of the disease is important. Laura Gomez-Virgilio and her collaborators, through a review of the literature, have evaluated the monitoring of oxidative stress by measuring NADH using the FLIM technique on olfactory neuron precursors, which had been isolated from patients in a non-invasive way [2]. This approach could allow researchers to find oxidative therapies in a more efficient way and then to personalize the follow-up of this disease.

Concerning Parkinson’s disease, the second most common neurodegenerative disorder, some authors have focused on a particular type of death, necroptosis, a cell death that is independent of the caspases but which involves receptor-interacting protein 3 (RIP3) and the pseudokinase mixed lineage domain-like protein (MLKL) or RIP1 kinase. Oliveira et al. identified a compound, Oxa12, that acts as an inhibitor of necroptosis not only in a BV-2 cell model when treated with the pan-caspase inhibitor zVAD-fmk, but also in vivo in a sub-acute 1-methyl-1-4-phenyl-1,2,3,6-tetrahydropyridine hydrochloride (MPTP) PD-related mouse model [3]. Another team targeted microglia-mediated neuroinflammation. They tested Epigallocatechin-3-gallate (EGCG), a natural antioxidant in green tea, by loading it into liposomes (phosphatidylcholine (PC) or phosphatidylserine (PS) coated with or without vitamin E) in an in vitro lipopolysaccharide (LPS)-induced BV-2 microglial cell activation model and in an in vivo model by targeting inflammation in the substantia nigra of Sprague-Dawley rats treated with LPS. The team of Cheng and collaborators was able to show that EGCG could inhibit inflammation and promote neuroprotection, making it a candidate for anti-Parkinson’s therapy [4].

In an effort to identify the molecules capable of reinforcing the available therapeutic arsenal, a team focused on multiple sclerosis and the evaluation of Apolipoprotein D protection in a cuprizone-induced cell model. The experiments that they carried out showed that the increase in Apolipoprotein D levels, whether exogenous or endogenous, moderately prevents the cytotoxic effects of cuprizone [5].

A study of repetitive mild traumatic brain injury (mTBI) showed that the TDP-43 proteinopathy identified in most cases of amyotrophic lateral sclerosis (ALS) did not impact the response in terms of chronic damage and inflammation in the optic tract [6].

More generally, different teams have highlighted the interrelationships between oxidative stress, cell death and inflammation in the context of neurodegenerative diseases [7] and have also compared these mechanisms with other pathologies, such as cancer or type 2 diabetes [8]. Others have targeted a specific mechanism, such as the team of Pluta et al., who chose to present the mechanisms involved in neuroinflammation in brain tissue after ischemia [9], or the team of Cadiele Oliana Reichert et al., who described the mechanisms of ferroptosis present in neurodegenerative diseases: lipid peroxidation, glutathione peroxidase 4 enzyme activity and iron metabolism [10].

We have seen that identifying a molecule as being able to inhibit the mechanisms responsible for neurodegeneration was not the only important step, as in the case of EGCG, but that the delivery method was also important. Karine Charrière and her collaborators from the Bio-PeroxIL Laboratory offered a summary of the known effects of docosahexaenoic acid (DHA), based on in vitro and in vivo targeting microglia and also based on clinical trials, while describing the nanomedicine techniques that have already been tested at the level of the microglia that could facilitate the action of DHA [11].

The editorial board would like to thank all the authors who participated in the success of this Special Issue by submitting quality articles. We hope that the articles published will help advance research on neurodegenerative diseases and that we will see the continuation of this work in the next special issue.
